# *Ac killer 1* establishes two epigenetically distinct regions associated with stable silencing of the maize *Ac* transposon

**DOI:** 10.3389/fpls.2026.1956494

**Published:** 2026-09-21

**Authors:** Dafang Wang, Sicong Wang, Lilly Horowitz, Meixia Zhao, Zuhair Ishraq, Charlotte Huang, Michelle Scelfo, Tejbir Singh, Ryan Singh, Sihun Kim

**Affiliations:** Hofstra University, Hempstead, NY, United States

**Keywords:** Ack1, Activator (Ac), maize, mop1, RNA-directed DNA methylation (RdDM), small RNAs, transposon silencing

## Abstract

**Introduction:**

We previously identified the silenced maize line *p1-ww-id1*, which carries *Ac killer 1* (*Ack1*), a naturally occurring inverted-repeat allele generated by alternative transposition. *Ack1* produces *Ac*-homologous small RNAs associated with reduced *Ac* activity, but how these small RNAs relate to *Ac* silencing across development remains unclear.

**Methods:**

We examined *Ack1*-associated silencing across four developmental stages representing embryonic, juvenile vegetative, adult vegetative, and reproductive tissues. *Ac* transcript abundance, region-specific small RNAs, and DNA methylation were analyzed, and *Ack1* was introduced into a *mop1* mutant background to test the contribution of the canonical 24-nt RNA-directed DNA methylation (RdDM) pathway.

**Results:**

Across all four developmental stages, the *Ac* subterminal/promoter region accumulated predominantly 24-nt small RNAs, whereas the Ac-transcribed region accumulated predominantly 21-22 nt small RNAs. Both regions gained CG, CHG, and CHH methylation despite their distinct small RNA compositions. *Ac* transcript abundance was reduced across the developmental series, with significant repression in embryonic, juvenile, and adult vegetative tissues. In V8 leaf 10, loss of MOP1 depleted subterminal/promoter 24-nt small RNAs and reduced DNA methylation in this region. In contrast, the *Ac*-transcribed region accumulated more 21–22-nt small RNAs and showed increased DNA methylation, while *Ac* remained transcriptionally repressed.

**Discussion:**

These findings reveal two epigenetically distinct regions of *Ac* characterized by different predominant small RNA populations and contrasting responses to disruption of canonical RdDM. The results support a model in which a naturally occurring inverted-repeat allele organizes region-specific epigenetic states across an active transposon while transcriptional repression persists.

## Introduction

The maize *Activator/Dissociation* (*Ac/Ds*) transposon system, first described by McClintock, is a classic model for DNA transposition and its effects on genome behavior (McClintock, 1950; Bennetzen and Wang, 2014). *Ac* is an autonomous hAT-family transposon that encodes the transposase required for excision and reintegration of both *Ac* and non-autonomous *Ds* elements (Fedoroff et al., 1983; Kunze and Weil, 2002). *Ac* and *Ds* share conserved terminal inverted repeats (TIRs) and adjacent subterminal regions. Both the 5′ and 3′ subterminal regions contain tandem AAACGG hexamer motifs that function as transposase-binding sites, and the 5′ subterminal region partially overlaps the *Ac* promoter (Kunze and Starlinger, 1989).

Although variation in *Ac* activity has been recognized for decades, the molecular basis of *Ac* silencing remains incompletely understood. Classical genetic studies identified two major phenomena associated with reduced *Ac* activity. The first is the negative dosage effect, in which increasing *Ac* copy number paradoxically suppresses transposition, although its mechanistic basis remains unresolved (McClintock, 1950, 1951). The second is DNA methylation within the AAACGG-containing transposase-binding sites of the subterminal/promoter region (Kunze and Starlinger, 1989; Wang et al., 1996; Becker and Kunze, 1997), which established an association between methylation of the subterminal/promoter region and reduced *Ac* activity, but did not determine how this methylation is induced, whether differential methylation is confined to the subterminal region or extends across *Ac*, or how methylation relates to small RNA pathways.

The discovery of small RNA-mediated epigenetic regulation transformed the understanding of transposon silencing in plants (Slotkin and Martienssen, 2007; Lisch, 2009; Matzke et al., 2015). In plants, RNA-directed DNA methylation (RdDM) is classically associated with a 24-nt small RNA pathway that requires RDR2, called MOP1 in maize (Alleman et al., 2006; Nobuta et al., 2008; Hale et al., 2009). In addition, 21- and 22-nt transposon-derived small RNAs can participate in non-canonical DNA methylation pathways (Nuthikattu et al., 2013; McCue et al., 2014; Cuerda-Gil and Slotkin, 2016; Burgess et al., 2020). A well-characterized maize example is *Mu killer* (*Muk*), a naturally occurring inverted-repeat allele that generates small RNAs and silences the *MuDR* transposon through epigenetic mechanisms involving RdDM-related pathways (Slotkin et al., 2003, 2005; Woodhouse et al., 2006; Burgess et al., 2020). These studies established that endogenous inverted-repeat alleles can generate transposon-homologous small RNAs and direct epigenetic silencing of active transposons.

A similar mechanism was later identified in the *Ac* system. Two silenced *Ac* lines, *p1-ww-id1* and *p1-ww-id4*, arose through sister-chromatid alternative transposition and each contains an inverted-repeat structure spanning the 5′ terminus of *Ac* into the coding region (Wang et al., 2020). These inverted repeats are transcribed to produce *Ac*-homologous double-stranded RNA and small RNAs that repress *Ac* transcript accumulation and *Ds* excision in trans (Wang et al., 2020). Because of their structural and functional similarity to *Muk*, these inverted-repeat alleles are collectively referred to here as *Ac killer* (*Ack*). *Ack* therefore represents the first naturally occurring inverted-repeat silencer of an hAT-family transposon described in maize. In this study, we focus on the *Ack* allele in *p1-ww-id1*, which we term *Ack1*; *Ack1* consists of an approximately 2,500 bp inverted repeat derived from the *Ac* 5′ region, exon 1 and the first half of exon 2, and is expressed from a nearby host-gene promoter (Wang et al., 2020).

Here, the *Ack1/Ac* system in *p1-ww-id1* was used to address three questions: whether *Ack1*-associated repression persists throughout maize development, whether *Ack1*-derived small RNAs are associated with region-specific DNA methylation across *Ac*, and whether different regions of *Ac* show distinct epigenetic responses to disruption of the canonical MOP1-dependent pathway. To address these questions, *Ac* transcript abundance, small RNA accumulation, and DNA methylation were examined across embryonic, juvenile vegetative, adult vegetative, and reproductive tissues, and *Ack1* was introduced into a *mop1* mutant background to perturb canonical RdDM. These analyses show that *Ack1*-mediated silencing is spatially organized rather than uniform across *Ac*, with two epigenetically distinct regions characterized by different predominant small RNA populations.

## Results

### *Ac* transcription is reduced throughout maize development in the presence of *Ack1*

*Ac* activity can be monitored using the *r1-m3* reporter, in which somatic excision of *Ds* restores anthocyanin pigmentation in the aleurone. In the progenitor line *p1-vv-9D9A*, active *Ac* promotes frequent transposition, producing coarse, early-onset purple sectors (**Figure 1A**). In contrast, the silenced line *p1-ww-id1* carries both a full-length *Ac* element and the naturally occurring *Ack1* allele, which generates *Ac*-homologous small RNAs and represses *Ac* activity (Wang et al., 2020). Consistent with this silenced state, *p1-ww-id1* kernels display delayed, fine spotting, indicative of reduced *Ac* transposase activity (**Figure 1A**).

**Figure 1:**
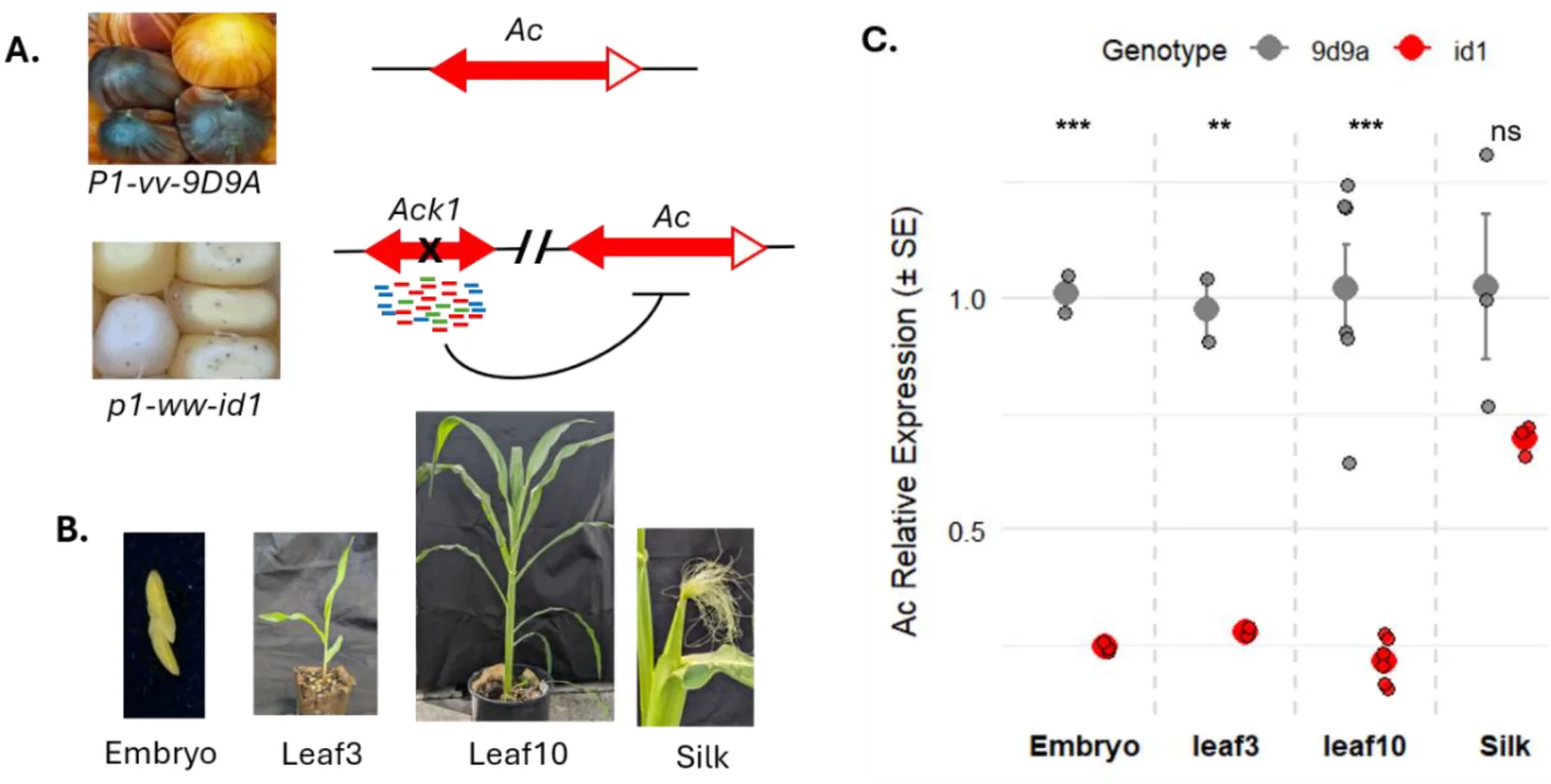
*Ack1*-associated transcriptional repression of *Ac* across maize developmental stages. **(A)** *Ac* silencing phenotype in maize aleurone. Representative kernels of the progenitor *p1-vv-9D9A* (active *Ac*) and the *Ack1*-containing silenced line *p1-ww-id1*. Active *Ac* promotes somatic *Ds* excision from the *r1-m3* reporter, producing coarse purple spotting, whereas *Ack1*-associated repression of *Ac* results in delayed excision and fine spotting. Schematics show the full-length *Ac* element and *Ack1* inverted repeat. Red arrows indicate *Ac* sequences; filled and open triangles indicate the 5′ and 3′ termini, respectively. Small RNAs are color-coded by size (blue, 24 nt; red, 22 nt; green, 21 nt); this color scheme is used throughout **Figures 2**-**6**. **(B)** Developmental stages analyzed: germinating embryos (41 h post-imbibition), V2 leaf 3, V8 leaf 10, and R1 silk. **(C)** Relative *Ac* transcript abundance determined by qRT-PCR using primers spanning exons 3–5, which are absent from *Ack1* and therefore specifically detect full-length *Ac* transcripts. Transcript abundance was normalized to *Ubiquitin* and is shown relative to *p1-vv-9D9A* within each tissue. Points represent biological replicates; large circles indicate means and error bars show ± SE. Statistical significance was assessed by Student’s *t*-tests on ΔCt values: *P < 0.05, **P < 0.01, ***P < 0.001; ns, not significant.

Our previous work established the structure of *Ack1* and demonstrated *Ac* repression in seedling tissues (Wang et al., 2020). Here, we quantified *Ac* transcript abundance in four developmental stages representing embryonic, juvenile vegetative, adult vegetative, and reproductive tissues: germinating embryos (41 h post-imbibition), V2 leaf 3, V8 leaf 10, and R1 silk (**Figure 1B**).

*Ac* transcript abundance was measured by qRT-PCR using primers spanning *Ac* exons 3–5, which lie outside the *Ack1* inverted repeat and therefore specifically detect full-length *Ac* transcripts (see Methods). Relative to the active progenitor *p1-vv-9D9A*, transcript abundance in *p1-ww-id1* was reduced by 75.6%, 71.3%, and 78.7% in embryos, V2 leaves, and V8 leaves, respectively (**Figure 1C**). These reductions were all statistically significant. In R1 silk, *Ac* transcript abundance was reduced by 32.1%, but this difference was not statistically significant. *Ack1*-associated small-RNA accumulation and regional *Ac* DNA methylation were nevertheless detected in silk (**Figures 2**, **3**). Raw Ct values, ΔCt and ΔΔCt calculations, relative expression values, and summary statistics are provided in **Supplementary Data 1**. Together, these results demonstrate strong *Ack1*-associated transcriptional repression in embryonic, juvenile, and adult vegetative tissues, with a more modest, nonsignificant reduction in R1 silk.

**Figure 2:**
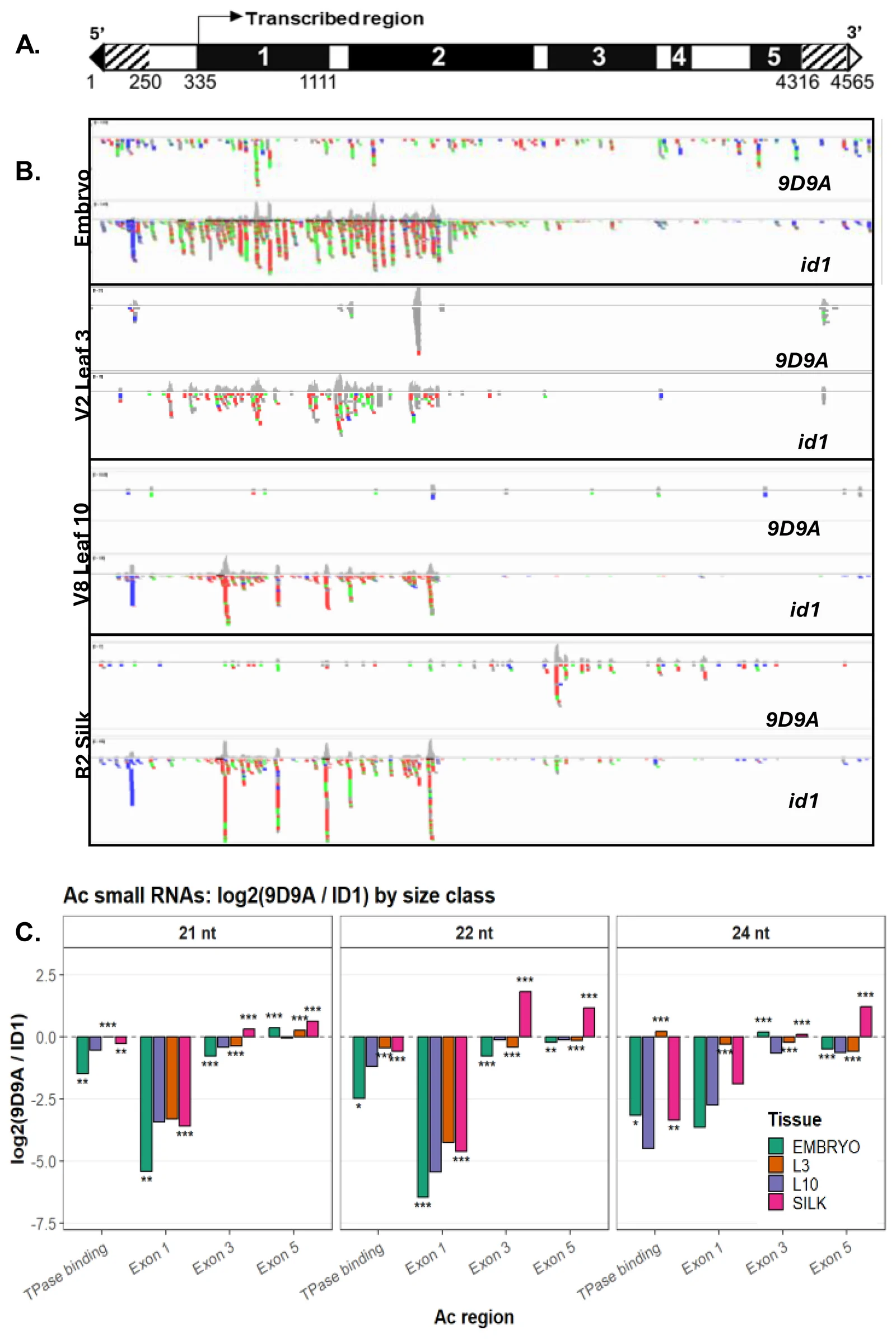
Region-specific distribution of *Ack1*-derived small RNAs across *Ac* during maize development. **(A)** Schematic of the *Ac* element showing the terminal inverted repeats (TIRs), the subterminal/promoter region (Sub-TIR), and the *Ac*-transcribed region. The numbers below the structure indicate the nucleotide position along *Ac*. **(B)** Genome browser visualization of *Ac*-derived small RNAs in germinating embryos, V2 leaf 3, V8 leaf 10, and R1 silk from *p1-vv-9D9A* and *p1-ww-id1*. Reads are color-coded by size as in **Figure 1A**, and multi-mapping reads were weighted according to mapping multiplicity. **(C)** Differential accumulation of 21-, 22-, and 24-nt small RNAs within the indicated *Ac* regions. Bars show log2(*p1-vv-9D9A*/*p1-ww-id1*) values; positive and negative values indicate enrichment in *p1-vv-9D9A* and *p1-ww-id1*, respectively. Statistical significance was assessed using a voom–limma model (Law et al., 2014). Asterisks indicate FDR-adjusted significance levels (*FDR < 0.05, **FDR < 0.01, ***FDR < 0.001).

**Figure 3:**
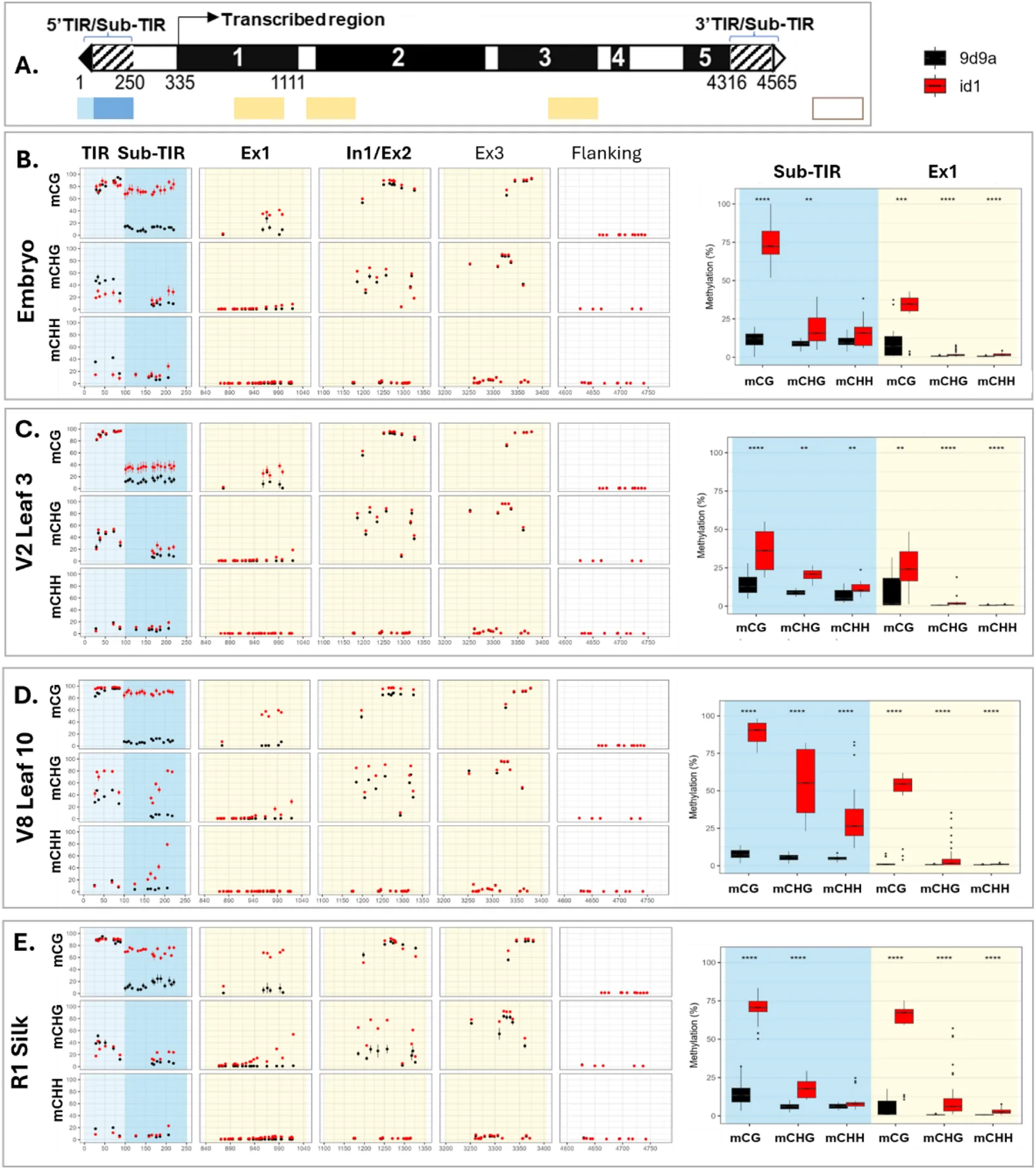
*Ack1*-associated DNA methylation across maize developmental stages. **(A)** Schematic of the *Ac* element (as in **Figure 2**) showing regions analyzed by targeted bisulfite sequencing. Colored boxes indicate the analyzed regions: light blue, 5′ TIR; blue, subterminal/promoter region; yellow, *Ac*-transcribed region and exon 3; unshaded, flanking region. **(B–E)** DNA methylation profiles in germinating embryos **(B)**, V2 leaf 3 **(C)**, V8 leaf 10 **(D)**, and R1 silk **(E)**. Left panels show mean DNA methylation (± SE) at individual cytosine positions across the regions shown in panel A for *p1-vv-9D9A* (black) and *p1-ww-id1* (red). Right panels compare DNA methylation within the subterminal/promoter region and exon 1 in CG, CHG, and CHH sequence contexts. Boxplots show the median, interquartile range, and 1.5 × IQR whiskers; each point represents one cytosine site averaged across three biological replicates (subterminal/promoter region, n = 28; exon 1, n = 55). Statistical significance was assessed using the Wilcoxon rank-sum test. Symbols indicate significance levels as defined in the figure * (P < 0.05), ** (P < 0.01), and *** (P < 0.001), ns (non-significant). Background colors match the colored boxes in **(A)**.

### Distinct small RNA populations accumulate in different regions of *Ac*

To determine whether developmentally persistent *Ac* repression is associated with the accumulation of *Ack1*-derived small RNAs, we sequenced small RNAs from the same four developmental stages in the active progenitor *p1-vv-9D9A* and the silenced genotype *p1-ww-id1* (**Supplementary Table 2**). Because terminal *Ac* sequences share similarity with hundreds of *Ds* elements in the maize genome, small RNA abundance was quantified using a weighted multi-mapping strategy in which each read contributes proportionally to all genomic matches (see Methods; **Supplementary Figure 1**; **Supplementary Data 2**).

Genome browser visualization revealed strong enrichment of small RNAs in *p1-ww-id1* across the *Ack1* inverted repeat, including the 5′ TIR, the subterminal/promoter region, exon 1, intron 1, and the first half of exon 2, whereas regions outside the *Ack1* inverted repeat, such as exon 3 and exon 5, showed comparatively little enrichment (**Figure 2B**; **Supplementary Data 2**). To quantify these regional differences, weighted 21-, 22-, and 24-nt small RNA counts were summarized over defined *Ac* regions and analyzed using a voom–limma model (Law et al., 2014) (**Supplementary Table 3**). The statistical analysis closely matched the browser visualization (**Figure 2C**). The subterminal/promoter region accumulated predominantly 24-nt small RNAs, whereas the *Ac*-transcribed region, represented by exon 1, accumulated predominantly 21- and 22-nt small RNAs. Exon 3 and exon 5 also showed increased small RNA abundance in *p1-ww-id1* in several tissues and size classes, but enrichment in these regions was substantially weaker and less consistent than in the *Ack1* inverted repeat region.

Although the overall regional organization was conserved, the magnitude of enrichment varied among developmental stages. In V2 leaf 3, enrichment of all three size classes within the subterminal/promoter region was weaker than in other tissues, whereas enrichment of 21- and 22-nt small RNAs within the *Ac*-transcribed region remained pronounced (**Figure 2C**; **Supplementary Table 3**). Consistent with this, V2 leaf 3 also exhibited the smallest DNA methylation differences between genotypes (**Figure 3C**), consistent with its weaker small RNA enrichment. Additional developmental features were also apparent. Region-specific peaks of 21–22-nt small RNAs became more pronounced in V8 leaves and R1 silk. Low-level 21–22-nt small RNAs were also detected immediately upstream of exon 1, particularly in embryos and V2 leaves (**Figure 2B**; **Supplementary Data 2**).

To confirm that these patterns were not dependent on the mapping reference, we repeated the analysis using an *Ac*-only reference while applying the same weighted multi-mapping and voom–limma pipeline. This analysis reproduced the principal features observed with the B73 reference, predominant enrichment of 24-nt small RNAs in the subterminal/promoter region and 21–22-nt small RNAs in the *Ac*-transcribed region, with differences only in absolute read counts (**Supplementary Figures 1**, **2**; **Supplementary Table 2**). These results demonstrate that the regional distribution of *Ack1*-derived small RNAs is robust to the choice of mapping reference.

### *Ack1*-associated silencing correlates with regional DNA methylation across *Ac*

To test whether the two region-specific small RNA populations are associated with corresponding DNA methylation patterns, we performed targeted bisulfite sequencing across six regions of the *Ac* element: the 5′ TIR, the subterminal/promoter region, three *Ac*-transcribed regions (exon 1, the intron 1/exon 2 region, and exon 3), and a flanking region outside *Ac* (**Figure 3A**). These regions include the major *Ack1*-associated small RNA peaks (5′ TIR, subterminal/promoter region, exon 1, and intron 1/exon 2) as well as exon 3 that show little *Ac*-derived small RNA enrichment and a non-homologous flanking segment. Summary statistics for each region and context are provided in **Supplementary Table 4**, and per-cytosine methylation values are listed in **Supplementary Data 3**.

In the progenitor *p1-vv-9D9A*, which reflects the methylation profile of an active *Ac* element, DNA methylation was distributed non-uniformly across *Ac*. CG methylation was relatively high within the 5′ TIR but declined sharply in the subterminal/promoter region, consistent with the high activity of this allele. Methylation remained low throughout the *Ac*-transcribed region before increasing again in downstream coding sequences, including exon 3, and was minimal in the flanking region. Thus, both the subterminal/promoter region and the *Ac*-transcribed region are relatively hypomethylated in the active state and are candidates for methylation changes associated with silencing.

In *p1-ww-id1*, the subterminal/promoter region exhibited the largest and most consistent methylation increases. Relative to *p1-vv-9D9A*, CG methylation increased substantially in all four developmental stages, with parallel increases in CHG and CHH methylation (**Figures 3B–E**). This region contains the AAACGG transposase-binding motifs previously implicated in methylation-sensitive regulation of *Ac* activity (Kunze and Starlinger, 1989; Wang et al., 1996; Becker and Kunze, 1997), indicating that *Ack1*-associated silencing is accompanied by increased DNA methylation at this regulatory region. Among tissues, V2 leaf 3 consistently showed the smallest increase, which is consistent with its weaker enrichment for *Ack1*-derived small RNAs.

A similar developmental pattern was observed in the *Ac*-transcribed region. Exon 1, which accumulated predominantly 21- and 22-nt small RNAs (**Figure 2**), showed significant increases in CG, CHG, and CHH methylation in *p1-ww-id1* relative to *p1-vv-9D9A* at all stages. As in the subterminal/promoter region, V2 leaf 3 displayed the smallest methylation increase. By contrast, exon 3 and the flanking region exhibited comparatively minor methylation differences between genotypes, in line with their limited small RNA enrichment and no *Ac* sequence homology, respectively.

Together, these results demonstrate that the two region-specific small RNA populations identified in **Figure 2** are associated with corresponding regional DNA methylation patterns across *Ac*. Both the subterminal/promoter region and the *Ac*-transcribed region acquire elevated CG, CHG, and CHH methylation despite their distinct small RNA compositions.

### Loss of MOP1 redistributes *Ack1*-derived small RNAs across *Ac* in V8 Leaf 10

Having identified two *Ac* regions with distinct small RNA populations associated with DNA methylation, we next examined how disruption of the canonical 24-nt small RNA pathway affects their regional distribution. To selectively disrupt the 24-nt population, we introduced the *p1-ww-id1* allele into a *mop1* mutant background, which impairs the RDR2-dependent pathway required for 24-nt small RNA biogenesis. The *mop1* analysis was performed in V8 leaf 10 using three biological replicates per genotype. This was expected to deplete 24-nt small RNAs from the subterminal/promoter region, enabling analysis of the remaining 21- and 22-nt species. A summary of the small RNA libraries and differential analyses is provided in **Supplementary Tables 2**, **3** and **Supplementary Data 2**.

Genome browser visualization indicated that loss of MOP1 substantially altered the regional distribution of *Ack1*-derived small RNAs (**Figure 4B**). In *p1-ww-id1;mop1/mop1*, 24-nt small RNAs were nearly absent from the subterminal/promoter region, whereas 21- and 22-nt small RNAs were enriched in the *Ac*-transcribed region. Because *mop1* causes a genome-wide reduction in 24-nt small RNAs, normalization by total library reads can alter relative abundance estimates of other size classes. We therefore used the miR156d/f/g-3p family, which was highly expressed and minimally affected by the *mop1* mutation, as an endogenous normalization reference (**Supplementary Figure 5**).

**Figure 4:**
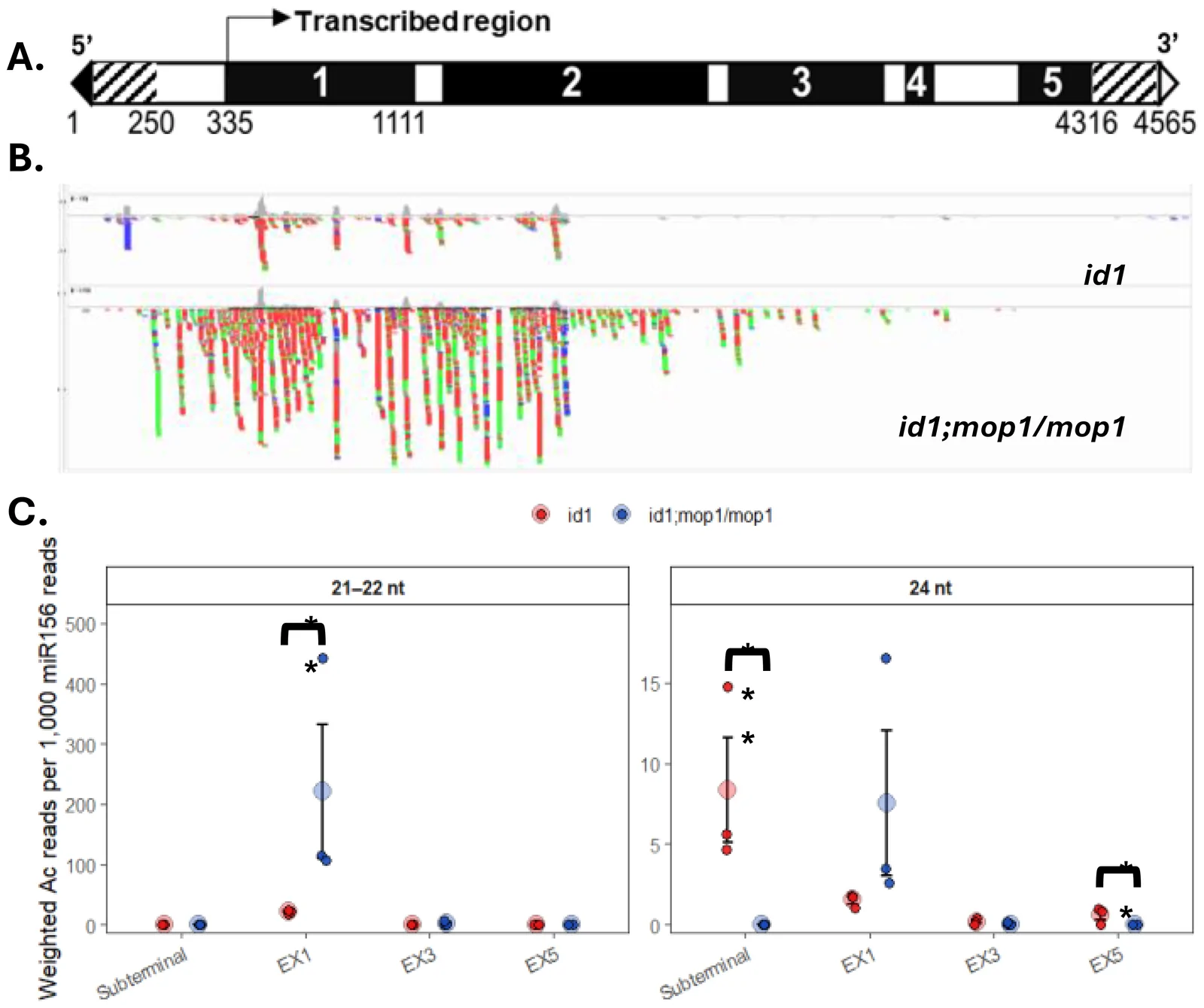
Loss of MOP1 redistributes *Ack1*-derived small RNAs across *Ac* in V8 leaf 10. **(A)** Schematic of the *Ac* element showing the subterminal/promoter region and the *Ac*-transcribed region. **(B)** Genome browser visualization of *Ac*-derived small RNAs in *p1-ww-id1* and *p1-ww-id1;mop1/mop1*. Reads are color-coded by size class (blue, 24 nt; red, 22 nt; green, 21 nt) and displayed in Integrative Genomics Viewer (IGV; Robinson et al., 2023) following weighted multi-mapping. Track positions correspond to the structural features shown in **(A, C)** Quantification of *Ac*-derived small RNAs within the indicated regions following normalization to endogenous miR156. Small RNA abundance is expressed as *Ac* reads per 1000 miR156 reads, with 21- and 22-nt size classes combined. Points represent biological replicates (n = 3 per genotype); large transparent circles indicate group means and error bars show ± SE. Asterisks indicate FDR-adjusted significance from an independent voom–limma analysis of the complete weighted small-RNA count dataset containing all three biological replicates per genotype. Significance levels are *FDR < 0.05, **FDR < 0.01, ***FDR < 0.001.

Using all three biological replicates per genotype, miR156-normalized abundance supported substantial and region-specific changes in *Ac*-derived small RNAs (**Figure 4C**). Within the subterminal/promoter region, mean 24-nt small RNA abundance decreased from 8.36 to 0.011 *Ac* reads per 1,000 miR156 reads in *p1-ww-id1;mop1/mop1*, an approximately 728-fold reduction. In contrast, exon 1 showed an approximately 10.3-fold increase in 21–22-nt small RNAs, from 21.60 to 221.47 *Ac* reads per 1,000 miR156 reads. The 21–22-nt population remained low in the subterminal/promoter region, while the increase in exon 1 24-nt small RNAs was more modest.

Differential abundance in **Figure 4C** was assessed using limma–voom on weighted small-RNA counts, with miR156 abundance used as the effective library size. This analysis included all three biological replicates per genotype, and p-values were adjusted across the eight displayed region × size-class comparisons using the Benjamini–Hochberg procedure. This analysis supported increased exon 1 21–22-nt small RNAs (FDR = 0.0059) and reduced subterminal-region 24-nt small RNAs (FDR = 1.29×10^−4^); exon 5 24-nt small RNAs were also reduced (FDR = 0.0049).

A total-read-normalized sensitivity analysis using all three biological replicates supported the same two principal changes: increased exon 1 21–22-nt small RNAs (FDR = 0.015) and reduced subterminal-region 24-nt small RNAs (FDR = $8.48\times 10^{-4}$) (**Supplementary Figure 4**). Thus, loss of MOP1 in V8 leaf 10 is associated with strong depletion of 24-nt small RNAs from the subterminal/promoter region and increased accumulation of 21–22-nt small RNAs within the *Ac*-transcribed region.

### MOP1 redistributes DNA methylation across distinct *Ac* regions while transcriptional repression persists in V8 Leaf 10

We next examined whether the MOP1-associated small RNA changes observed in V8 leaf 10 were accompanied by corresponding changes in DNA methylation and *Ac* transcription. Within the subterminal/promoter region, loss of MOP1 significantly reduced DNA methylation in all three sequence contexts relative to *p1-ww-id1*. CHG and CHH methylation showed the largest decreases, whereas CG methylation declined more modestly, although it is still significantly lower than in *p1-ww-id1*. However, in the *mop1* mutant, methylation in all contexts remains significantly higher than in *p1-vv-9D9A* (**Figures 5B, C**; **Supplementary Table 4**). These patterns indicate that the MOP1-dependent RdDM pathway makes a major contribution to methylation of this regulatory region but is not solely responsible for maintaining its highly methylated state. The *Ac*-transcribed region exhibited the opposite response. Exon 1, which accumulated markedly higher levels of 21- and 22-nt small RNAs in the *mop1* background, also showed significantly increased CG, CHG, and CHH methylation relative to *p1-ww-id1*, with the largest increase in the CG context.

**Figure 5:**
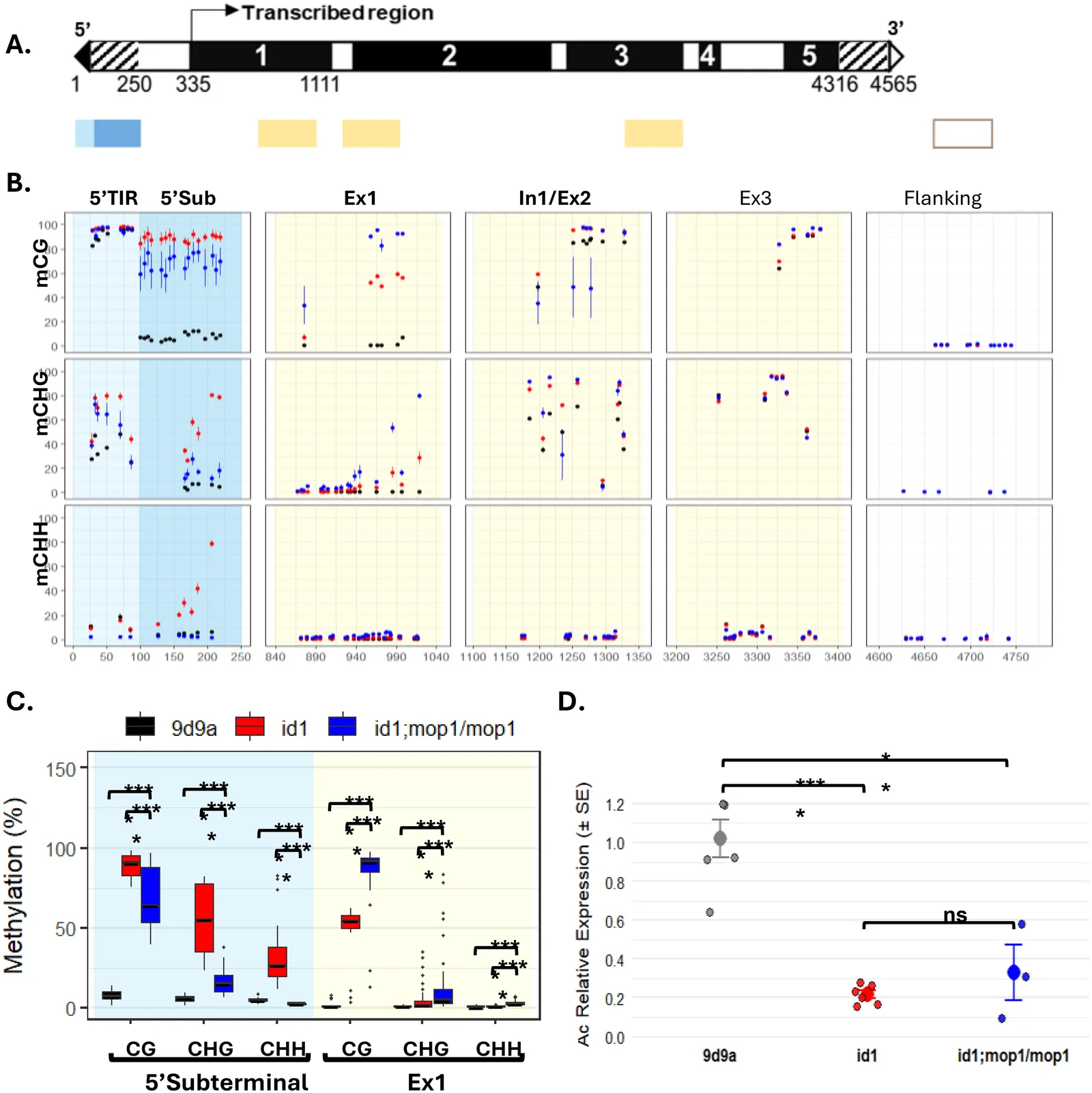
Loss of MOP1 differentially affects DNA methylation across distinct *Ac* regions while *Ac* remains transcriptionally repressed in V8 leaf 10. **(A)** Schematic of the *Ac* element showing the regions analyzed by targeted bisulfite sequencing, as defined in **Figure 3**. **(B)** DNA methylation profiles of individual cytosine positions across the indicated *Ac* regions in *p1-vv-9D9A* (black), *p1-ww-id1* (red), and *p1-ww-id1;mop1/mop1* (blue). Each point represents the methylation level of one cytosine site averaged across sequencing reads and three biological replicates; error bars indicate ± SE. **(C)** Quantification of DNA methylation within the subterminal/promoter region and exon 1 in CG, CHG, and CHH sequence contexts. Boxplots show the median, interquartile range, and 1.5 × IQR whiskers. Statistical significance was assessed using Wilcoxon rank-sum tests; complete pairwise comparisons are provided in **Supplementary Table 4**. Significance levels are indicated as *P* < 0.05 (*), P < 0.01 (**), P < 0.001 (***); P < 0.0001 (****); ns, not significant. **(D)** Relative *Ac* transcript abundance determined by qRT-PCR using primers spanning *Ac* exons 3–5 and normalized to Ubiquitin. Points represent biological replicates (n = 6 for *p1-vv-9D9A* and *p1-ww-id1*; n = 3 for *p1-ww-id1;mop1/mop1*); large circles indicate group means and error bars represent ± SE. Statistical significance was assessed using Student’s t-tests on ΔCt values; ns, not significant; * P<0.05.

To determine whether these contrasting regional methylation responses altered *Ac* transcription, we quantified *Ac* transcript abundance using the same qRT-PCR assay described in **Figure 1**. In *p1-ww-id1;mop1/mop1*, transcript abundance was significantly lower than in *p1-vv-9D9A* and similar to that of *p1-ww-id1* (**Figure 5D**; **Supplementary Data 1**), indicating that disruption of MOP1 does not restore *Ac* transcription despite extensive remodeling of regional small RNAs and DNA methylation.

Together, these results show that loss of the MOP1-dependent RdDM pathway is associated with contrasting epigenetic changes in the two *Ac* regions while *Ac* remains transcriptionally repressed. Although increased small RNA accumulation and DNA methylation in the *Ac*-transcribed region coincide with reduced 24-nt small RNAs and methylation in the subterminal/promoter region, the present data do not establish a functional or compensatory relationship between these responses.

### A working model for *Ack1*-mediated epigenetic silencing of Ac

The experiments presented here support a working model in which *Ack1* partitions *Ac* into two epigenetically distinct regions that differ in their predominant small RNA populations and in their responses to disruption of the MOP1-dependent RdDM pathway (**Figure 6**). Rather than behaving as a uniformly regulated silencing locus, *Ac* exhibits two region-specific epigenetic patterns associated with transcriptional repression.

**Figure 6:**
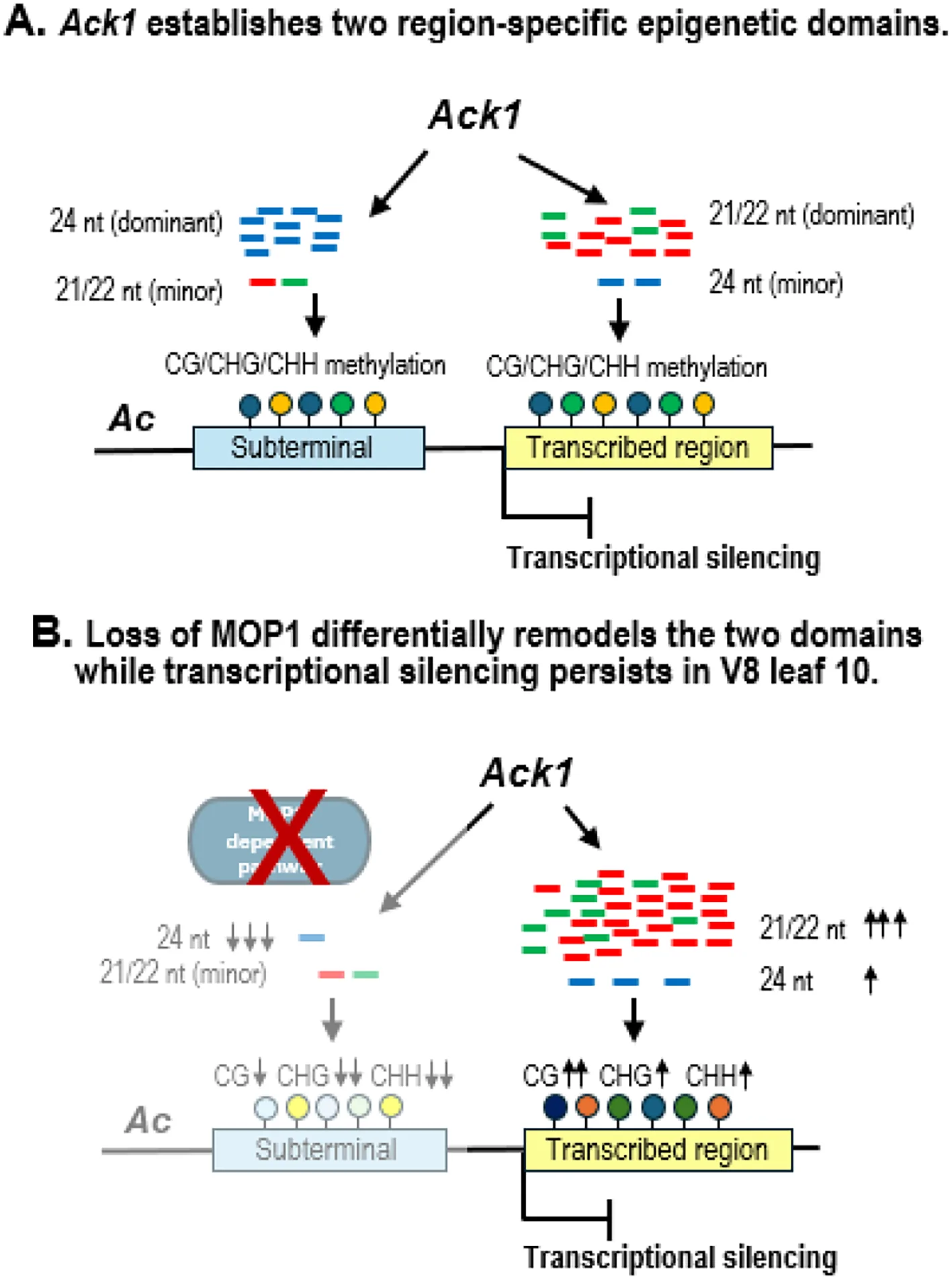
Working model of *Ack1*-mediated epigenetic silencing of *Ac*. **(A)** *Ack1*-associated DNA methylation under wild-type conditions. *Ack1* partitions *Ac* into two epigenetically distinct regions with different predominant small RNA populations. The subterminal/promoter region is enriched mainly for 24-nt small RNAs with lower levels of 21–22-nt small RNAs, whereas the *Ac*-transcribed region is enriched mainly for 21–22-nt small RNAs with lower levels of 24-nt small RNAs. Both regions show CG, CHG, and CHH DNA methylation and are associated with transcriptional repression of *Ac*. **(B)** Working model following loss of MOP1 in V8 leaf 10. Disruption of the canonical RdDM pathway strongly reduces 24-nt small RNAs in the subterminal/promoter region, accompanied by decreases in CG, CHG, and CHH methylation. In contrast, the *Ac*-transcribed region accumulates substantially higher levels of 21–22-nt small RNAs, and exhibits increased CG, CHG, and CHH methylation. Despite these contrasting regional responses, *Ac* remains transcriptionally repressed. Because *mop1* was analyzed only at this developmental stage, the MOP1-dependent remodeling illustrated here should not be extrapolated to other developmental stages.

The subterminal/promoter region corresponds to the classically characterized regulatory region containing the *Ac* promoter and transposase-binding sites. This region is enriched mainly for 24-nt small RNAs and, in V8 leaf 10, responds to loss of MOP1 with reduced DNA methylation, particularly in the CHG and CHH contexts. By contrast, the *Ac*-transcribed region is enriched mainly for 21- and 22-nt small RNAs and, at the same developmental stage, responds to loss of MOP1 with increased DNA methylation.

A notable outcome of this regional specialization is that extensive remodeling of both small RNA populations and DNA methylation does not lead to transcriptional reactivation of *Ac*. Instead, *Ac* remains transcriptionally repressed following loss of MOP1, indicating that the RdDM pathway contributes substantially to methylation within the subterminal/promoter region but is not solely responsible for *Ack1*-associated repression. Because *mop1* was examined only in V8 leaf 10, whether similar regional remodeling occurs during embryonic, juvenile vegetative, or reproductive development remains unknown.

This model integrates the observed region-specific responses to disruption of MOP1 while distinguishing these observations from their possible mechanistic relationship. Whether the contrasting responses of the two regions are functionally related remains unknown. The mechanistic basis of these regional differences, including the roles of MOP1-dependent amplification and potential non-canonical small RNA pathways, remains to be determined.

## Discussion

### *Ack1* expands the known methylation landscape of *Ac*

The DNA methylation target previously implicated in regulating *Ac* activity is located in the subterminal region, where methylation of AAACGG transposase-binding motifs has long been associated with reduced *Ac* activity and transposition (Chomet et al., 1987; Kunze and Starlinger, 1989; Brutnell and Dellaporta, 1994; Wang et al., 1996; Becker and Kunze, 1997; Ros and Kunze, 2001). Earlier studies relied on methylation-sensitive restriction enzymes, whereas our bisulfite sequencing provides a high-resolution DNA methylation landscape across the entire 5′ subterminal region in all cytosine contexts. Consistent with previous reports, the silenced allele exhibited increased methylation within this region.

Our results further demonstrate that differential methylation is not restricted to these promoter-proximal regulatory sequences. The *Ack1*-transcribed region, encompassing exon 1 and the intron 1/exon 2 region, is weakly methylated in the active *Ac* line *p1-vv-9D9A* but acquires substantial CG, CHG, and CHH methylation in the silenced line *p1-ww-id1*, and is enriched predominantly for 21–22-nt small RNAs. In contrast, exons such as exon 3, which are already highly methylated in the active line, change comparatively little. Thus, *Ack1*-associated silencing is accompanied by differential methylation across multiple regions of *Ac* rather than being restricted to the historically characterized subterminal regulatory motifs.

### The MOP1-dependent pathway contributes to *Ack1*-derived 24-nt small-RNA accumulation

Our previous study showed that the *Ack1* inverted repeat in intron 1 of Zm00001d028930 produces double-stranded RNA from a host-gene promoter rather than the native *Ac* promoter (Wang et al., 2020). This dsRNA is detected in roots, where the promoter is active, but not in shoots, where it is inactive. In the present study, *Ack1*-derived small RNAs were detected across developmental stages, but primary dsRNA was not measured. Therefore, whether the small RNAs observed in these tissues derive directly from host-gene-driven *Ack1* transcripts or from secondary RNA-dependent amplification cannot be determined from the present data.

Our *mop1* analysis provides evidence that MOP1 contributes to the accumulation of the 24-nt small RNA population in V8 leaf 10. Loss of MOP1 strongly reduced 24-nt small RNAs in the subterminal/promoter region and was accompanied by reduced CHG and CHH methylation. Because MOP1 is the maize RDR2 homolog, these results indicate that accumulation of the abundant subterminal-region 24-nt small-RNA population in V8 leaf 10 depends substantially on the MOP1/RDR2 pathway. One possible model, consistent with the amplification hypothesis proposed in our previous study (Wang et al., 2020), is that primary *Ack1*-derived RNA initiates small RNA production, whereas MOP1/RDR2 subsequently reinforces the 24-nt population. However, because primary and secondary dsRNA precursors were not distinguished experimentally, secondary amplification remains a working hypothesis rather than a direct conclusion of this study.A distinct response was observed in the *Ac*-transcribed region, where 21–22-nt small RNAs increased following loss of MOP1. This observation raises the possibility that an additional small RNA biogenesis pathway contributes to this population. RDR6 is one candidate because it generates 21–22-nt transposon-derived small RNAs in Arabidopsis (Nuthikattu et al., 2013; McCue et al., 2014). However, RDR6 involvement was not tested directly, and the relationship between depletion of the MOP1-dependent 24-nt population in the subterminal/promoter region and increased 21–22-nt small RNAs in the *Ac*-transcribed region remains unresolved. Analysis of *rdr6* mutants and developmental-stage-specific characterization of *Ack1*-derived dsRNA will be required to distinguish among these possibilities.

Despite extensive remodeling of small RNA populations and DNA methylation, *Ac* transcription remained strongly repressed. Thus, disruption of canonical RdDM alters the epigenetic landscape of *Ac* without reactivation. Whether the increased small RNA accumulation and methylation in the *Ac*-transcribed region contributes functionally to the persistence of repression remains to be determined.

### Implications for alternative-transposition-induced silencing

*Ack1* is a naturally occurring example showing that alternative transposition can create a long-lasting epigenetic silencer. Building on our previous work that alternative transposition can start *Ac* silencing (Wang et al., 2020), this study shows that the silenced state is maintained across development and is linked to region-specific small RNA populations and DNA methylation along the transposon.

The two-region organization we observe is similar to patterns seen in other plant transposon-silencing systems, such as *Muk*-mediated silencing of *MuDR*, where different regions of the element are targeted by distinct small RNA populations and RdDM pathways (Burgess et al., 2020).

Overall, our results support a model in which a naturally occurring inverted-repeat allele organizes its target transposon into two epigenetically distinct regions characterized by different predominant small RNA classes and distinct responses to disruption of canonical RdDM, while *Ac* remains transcriptionally repressed. This refines current models of alternative-transposition-induced silencing and provides a framework for understanding how inverted-repeat alleles establish durable epigenetic repression in plants. Because the *mop1* analyses were restricted to V8 leaf 10, the MOP1-dependent remodeling described here should be interpreted as stage-specific. Whether similar responses occur at other developmental stages remains unknown and will require additional genetic analyses.

## Materials and methods

### Plant materials and tissue collection

The maize lines *p1-vv-9D9A* (active *Ac*), *p1-ww-id1* (containing both *Ack1* and a full-length *Ac* element), and *p1-ww-id1; mop1/mop1* (Lisch et al., 2002) were grown under standard greenhouse conditions (25–30 °C). Seeds were germinated in potting mix and transplanted to larger pots at approximately the V2 developmental stage.

Tissues were collected at four developmental stages: germinating embryos (41 h post-imbibition), juvenile vegetative leaves (V2, leaf 3), adult vegetative leaves (V8, leaf 10), and reproductive tissue (R1 silks). Samples were flash-frozen in liquid nitrogen and stored at −80 °C until use. Unless otherwise indicated, three biological replicates (independent plants) were analyzed for each genotype × tissue combination.

The *r1-m3* reporter background was not tracked during generation of the *p1-ww-id1;mop1/mop1* material; therefore, kernel spotting was not used to assess *Ac* activity in the *mop1* background.

### Genotyping

The *p1-vv-9D9A*, *p1-ww-id1*, and *mop1* genotypes were confirmed by PCR using primers listed in **Supplementary Table 1**. Homozygosity of the *mop1* allele was confirmed for all plants used in the *mop1* analyses.

### RNA extraction

Total RNA was extracted from frozen tissues using the Direct-zol RNA Miniprep Plus Kit (Zymo Research) with on-column DNase treatment, following the manufacturer’s instructions. RNA concentration was measured using the Qubit RNA Broad Range Quantification Assay (Thermo Fisher Scientific), and RNA integrity was evaluated using the Qubit RNA Integrity and Quality Assay (Thermo Fisher Scientific).

### Quantitative RT-PCR

Quantitative RT-PCR was performed using the Luna Universal One-Step RT-qPCR Kit (New England Biolabs) on a Bio-Rad CFX Connect Real-Time PCR Detection System. *Ac* transcript abundance was measured using primers spanning *Ac* exons 3–5, which lie outside the *Ack1* inverted repeat and therefore specifically detect full-length *Ac* transcripts. Ubiquitin (GRMZM2G409726) served as the internal reference gene. Primer sequences are provided in **Supplementary Table 1**.

Relative transcript abundance was calculated using the 2^-ΔΔCt^ method (Livak and Schmittgen, 2001) for visualization, and statistical comparisons between genotypes were performed using Student’s t-tests on ΔCt values (Yuan et al., 2006).

### Small RNA library construction and sequencing

Small RNA libraries were prepared from germinating embryos, V2 leaf 3, V8 leaf 10, and R1 silk tissues by CD Genomics (Shirley, NY) using gel-based size selection of 18–30 nt inserts and sequenced on an Illumina NovaSeq platform. Three biological replicates were analyzed for each genotype × tissue combination.

### Small RNA processing and alignment

Adapter-trimmed small RNA reads were aligned to the maize B73 RefGen_v5 assembly using Bowtie v1 (Langmead et al., 2009) with parameters -v 0 -a --best --strata, allowing zero mismatches and reporting all equally best alignments.

The *Ac* element (GenBank accession X05424) was manually annotated in GFF3 format, including the 5′ terminal inverted repeat (TIR), subterminal/promoter region, *Ac*-transcribed region (exon 1 and the intron 1/exon 2 region), exon 3, exon 5, and a flanking region outside *Ac*. Genome browser visualization was performed using Integrative Genomics Viewer (IGV) (Robinson et al., 2011, 2023).

### Weighted quantification of *Ac*-derived small RNAs

Because terminal *Ac* sequences share homology with other *Ds* elements in the maize genome, restricting to uniquely mapped reads would underestimate regional small RNA abundance. We therefore adopted a weighted multi-mapping strategy following approaches developed for transposon-derived small RNAs.

Each read was assigned a weight of 1/n, where n is the number of equally best genomic alignments, such that each small RNA contributes a total weight of one while retaining proportional representation at repetitive loci. Weighted read counts were summarized for each annotated *Ac* feature and stratified by small RNA size class (21, 22, and 24 nt).

To verify that the observed regional enrichment patterns were not dependent on the reference genome, all analyses were repeated using an *Ac*-only reference sequence while applying the same alignment, weighting, normalization, and statistical procedures.

### Normalization of small RNA abundance

Because loss of *MOP1* causes a genome-wide reduction in 24-nt small RNAs, normalization to total mapped reads can distort relative abundance estimates. We therefore evaluated candidate endogenous miRNAs as normalization references by comparing their abundance, coefficient of variation, and differential expression between *p1-ww-id1* and *p1-ww-id1; mop1/mop1* libraries.

The miR156d/f/g-3p family exhibited high abundance, low variation, and no significant differential expression and was therefore selected as the normalization reference. *Ac*-derived small RNA abundance was normalized to miR156 abundance. Summary statistics for candidate reference miRNAs are provided in **Supplementary Figure 5**.

### Targeted bisulfite sequencing

Genomic DNA was extracted using the Plant DNA Extraction Kit (Zymo Research). Bisulfite conversion, PCR amplification, and library preparation were performed by CD Genomics (Shirley, NY).

Six regions of *Ac* were amplified following bisulfite conversion: the 5′ TIR, subterminal/promoter region, *Ac*-transcribed region (exon 1 and the intron 1/exon 2 region, exon 3), and a flanking region outside *Ac*. Primer sequences are provided in **Supplementary Table 1**.

Bisulfite reads were trimmed and aligned to the *Ac* reference sequence using Trimmomatic v0.36 and BSMAP v2.73. Methylation levels were calculated separately for CG, CHG, and CHH contexts at each cytosine using the BSMAP methylation extractor. Only cytosines covered by ≥20 reads were retained for downstream analysis.

### Statistical analysis

Differential abundance of Ac-derived small RNAs was analyzed using the limma–voom framework (Law et al., 2014; Ritchie et al., 2015) on weighted read-count matrices. For developmental comparisons in **Figure 2**, library-size normalization was performed within the edgeR/limma–voom workflow, and Benjamini–Hochberg-adjusted *P*values were used to control the false discovery rate.

For the V8 leaf 10 *mop1* comparison in **Figure 4C**, weighted Ac-derived small-RNA counts were analyzed by limma–voom using miR156d/f/g-3p abundance as the effective library size. miR156-normalized values were used for visualization and fold-change estimates. *P*values were adjusted by the Benjamini–Hochberg procedure across the eight plotted region × size-class comparisons. A total-read-normalized limma–voom sensitivity analysis was performed using total library reads as effective library sizes and is shown in **Supplementary Figure 4**.

Regional differences in DNA methylation between genotypes were evaluated using Wilcoxon rank-sum tests on per-cytosine methylation values, following approaches commonly used for targeted bisulfite amplicon analyses in maize.

## Data Availability

Small RNA sequencing and targeted bisulfite sequencing datasets generated in this study have been deposited in the NCBI Sequence Read Archive under BioProject accession PRJNA1506355.

## Supplementary material

Supplementary Figure 1 Workflow of weighted multi-mapping analysis. Overview of the computational workflow used for weighted multi-mapping, region-level quantification, and differential analysis of Ac-derived small RNAs.

Supplementary Figure 2 Ac-only reference mapping of small RNAs. Browser visualization of Ac-derived small RNAs mapped to an Ac-only reference sequence, demonstrating that the regional distribution of Ack1-derived small RNAs is independent of the mapping reference.

Supplementary Figure 3 Quantification of Ac-only reference mapping. Quantitative comparison of Ac-derived 21-, 22-, and 24-nt small RNAs mapped to the Ac-only reference, confirming the regional enrichment patterns observed using the B73+Ac reference.

Supplementary Figure 4 Total-read normalization of Ac-derived small RNAs in *mop1* mutant. Quantification of Ac-derived small RNAs using total mapped read normalization, demonstrating that the regional redistribution observed in the mop1 mutant is consistent with the miR156-normalized analysis presented in the main text.

Supplementary Figure 5 Selection of miR156 as the normalization reference. Evaluation of candidate endogenous miRNAs based on abundance, biological variation, and genotype effects, supporting the selection of miR156 as a stable normalization reference.

Supplementary Table 1 Primer sequences and amplicon information. Primer sequences, target regions, and amplicon information used for genotyping, qRT-PCR, and targeted bisulfite sequencing.

Supplementary Table 2 Small RNA sequencing library summary. Summary statistics for each small RNA sequencing library, including sequencing depth, mapping statistics, and total weighted Ac-derived small RNA counts.

Supplementary Table 3 Complete voom–limma differential small RNA analysis. Complete statistical results from voom–limma analysis of Ac-derived small RNAs across developmental stages, genomic regions, size classes, and genotype comparisons.

Supplementary Table 4 DNA methylation summary statistics. Summary statistics for DNA methylation analyses, including mean methylation levels, sample sizes, and Wilcoxon rank-sum test results for all genomic regions and sequence contexts.

Supplementary Table 5 Experimental design summary. Overview of developmental stages, tissues, genotypes, assays, biological replicates, and corresponding main figures included in this study.

Supplementary Table 6 Annotation of Ac genomic regions. Coordinates and annotations of Ac genomic regions analyzed for small RNA profiling and targeted bisulfite sequencing.

Supplementary Data Sheet 1 Complete qRT-PCR calculations. Raw Ct values, ΔCt, ΔΔCt, relative transcript abundance, and statistical analyses for all qRT-PCR experiments.

Supplementary Data Sheet 2 Complete weighted Ac-derived small RNA counts. Weighted read counts for all Ac genomic regions, small RNA size classes, biological replicates, and developmental stages used in downstream analyses.

Supplementary Data Sheet 3 Per-cytosine DNA methylation data. DNA methylation values for every cytosine analyzed across all developmental stages, genotypes, and sequence contexts.
